# Incidence and Risk Factors of Venous Thromboembolism in Patients After Transurethral Resection of the Prostate (TURP)

**DOI:** 10.3389/fsurg.2021.744244

**Published:** 2022-02-07

**Authors:** Zhihuan Zheng, Ziqiang Wu, Kaixuan Li, Quan Zhu, Haozhen Li, Xuesong Liu, Guilin Wang, Zhengyan Tang, Zhao Wang

**Affiliations:** ^1^Xiangya Hospital, Central South University, Changsha, China; ^2^Provincial Laboratory for Diagnosis and Treatment of Genitourinary System Disease, Changsha, China

**Keywords:** benign prostatic hyperplasia, incidence, risk factors, venous thromboembolism, TURP

## Abstract

**Objective:**

Venous thromboembolism (VTE) in patients following transurethral resection of the prostate (TURP) has been overlooked for many years. This research was aimed to investigate the incidence and risk factors of VTE in patients after TURP.

**Methods:**

A total of 451 patients who underwent TURP between January 2017 and December 2020 were retrospectively analyzed. Clinical data of the patients were collected, such as basic demographic data, prostate volume, creatinine values, hemoglobin values, surgery duration, Caprini score, international prostate symptom score (IPSS), quality of life (QOL) score, plasma D-dimer levels, and so on. Univariate analysis and multivariate logistic regression were performed to identify the potential risk factors of VTE. Venous ultrasonography of lower extremities was performed routinely to detect VTE for patients after TURP.

**Results:**

In total, 36 (8%) out of the 451 patients suffered from VTE. A total of 12 (2.7%) patients were confirmed with deep venous thrombosis (DVT). Two patients (0.4%) were identified with pulmonary embolism (PE). Twenty-two (4.9%) patients were suffered from superficial venous thrombosis. Furthermore, according to the results of multivariate stepwise logistic regression analysis, having a history of VTE (adjusted odds ratio [aOR] = 10.980, 95% CI = 2.265–53.223), complicated with postoperative bladder hematoma (aOR = 6.302, 95% CI = 2.265–17.532), D-dimer >1.25 mg/L (aOR = 4.402, 95% CI = 1.798–10.774), and age >65 (aOR = 3.106, 95% CI = 1.084–8.893) were independent risk factors of VTE after TURP. In addition, the nomogram prediction model is a useful auxiliary prevention tool of VTE.

**Conclusion:**

The incidence of VTE is severely underestimated in patients following TURP. A lot of asymptomatic VTEs have been overlooked. Early detection and diagnosis of VTE are essential. Nevertheless, further verifications based on the results of large-scaled studies are still needed.

## Introduction

Benign prostatic hyperplasia (BPH) is one of the most common benign diseases causing dysuria in elderly men. Approximately, 50% of men over 50 years have evidence of BPH and by the time they were 80 years old, the proportion of men with BPH increases to 80% (1). For decades, the gold standard for surgical treatment of BPH remains transurethral resection of the prostate (TURP). While, this procedure carries the risk of bleeding, clot retention, bladder neck contracture or urethral stricture, urinary incontinence, erectile dysfunction, and retrograde ejaculation (2). In clinical practice, urologists are more concerned about the abovementioned risks, but little attention has been paid to the risk of venous thromboembolism (VTE). VTE, which consists of vein thrombosis (DVT) and pulmonary embolism (PE), was a potentially fatal complication after surgery (3). Thirty-day mortality of the VTE patients can reach more than 10-fold of the non-VTE patients (4) and about 30% of patients with PE die within the first year after diagnosis (5). Recent studies on VTE in urological non-oncological patients demonstrate that the incidence of VTE is not as rare as previously reported (6–9). Few studies have been concerned about the incidence and risk factors of VTE in patients following TURP. So, in this study, we aimed to retrospectively investigate the incidence and risk factors of VTE in recent years in our institution after TURP.

## Methods

### Patients

We retrospectively collected the clinical data of patients who underwent TURP in our institution, a tertiary hospital with the highest level of referral directly under the National Healthy Committee of China. The data were obtained through Electronic Medical Record System (EMRS). The inclusion criteria were as follows: (1) patients who were eligible for TURP from January 2017 to December 2020; (2) more than 18 years of age. The following were exclusion criteria: (1) complicated with active malignant disease; (2) having a history of prostate surgery or urinary reconstructive surgery; (3) postoperative pathological examination revealing prostate cancer; (4) physical condition was inappropriate to perform TURP (such as poor cardiopulmonary function and poor coagulation function); and (5) VTE was found preoperatively in the lower extremities by ultrasound.

### Thrombosis Prevention

The risk of VTE of the inpatients was assessed by the Caprini score when admission. If the patients were intermediate-risk or high-risk, mechanical thromboprophylaxis (graduated compression stockings and intermittent pneumatic compression device) was used after surgery. Pharmacological prophylaxis was not regularly performed according to the recommendation of the European Association of Urology (EAU) guidelines and the American Society of Hematology guidelines (10, 11).

### Diagnosis and Treatment of VTE

Ultrasound for bilateral lower extremities was performed preoperatively and postoperatively in intermediate-risk and high-risk patients. Incompressibility intravascular defects or absences of phase Doppler signal were identified as positive of VTE (12). The diagnosis of PE was established by the VTE group in our institution according to either computed tomographic pulmonary arteriography (CTPA), echocardiography or suspicious symptoms of PE, such as dyspnea, palpitation, and/or pleuritic chest pain developed after surgery. Postoperative hemoglobin and plasma D-dimer concentration were measured within 24 h after surgery. Other biochemical indicators were measured within 24 h after admission. Once the patient was diagnosed with VTE, the mechanical thromboprophylaxis was quit immediately. Then, the patient will be treated under the guidance of interdisciplinary consultation. Three months of anticoagulant with rivaroxaban was maintained after discharge according to the actual condition. Vascular ultrasonography would be re-performed 3 months later.

### Data Analysis

Categorical variables were presented as percentages (%) and analyzed using the chi-squared or Fisher's exact tests, as appropriate. Quantitative data were presented as mean ± SD or median (interquartile intervals) and analyzed by Student's *t* or the Mann-Whitney U test, as appropriate. Univariate, multivariate logistic regression models, and multivariate stepwise logistic regression models were performed to identify the risk factors for VTE after TURP. A two-tailed *p* <0.05 was considered statistically significant. All statistical analyses were performed by IBM SPSS Statistics for Windows, version 25.0 (IBM Corp, Armonk, NY, USA) and R version 4.1.0.

## Results

### Incidence

A total of 451 patients who underwent TURP were enrolled in the study. There were 8.0% (36/451) patients found with VTE. In total, 2.7% (12/451) of the patients were diagnosed with DVT and 0.4% (2/451) of the patients were confirmed with PE. In addition, 4.9% (22/451) of the patients were suffered from superficial vein thrombosis such as calf intermuscular vein thrombosis (Table 1).

**Table 1:** Baseline demographic and clinical characteristics.

**Variables**	**Total patients**	**VTE**	**Non-VTE**	***P*-value**
Count	451	36	415	–
Age (years), median (IQR)	68 (64–74)	70 (68–77)	68 (64–73)	**0.049**
BMI (kg/m2), mean ± SD	23.47 ± 3.05	22.71 ± 2.18	23.54 ± 3.12	0.124
Prostate volume (ml), median (IQR)	52.20 (39.48–70.90)	55.80 (42.70–76.40)	52.85 (38.98–73.43)	0.857
Creatinine (μmol/L), median (IQR)	87.0 (79.2–97.0)	93.6 (82.0–103.0)	86.9 (79.0–95.0)	0.116
**Hb (g/L), median (IQR)**				
Preoperative	137 (128–145)	133 (128.75–145.00)	136 (122.00–144.25)	0.361
Postoperative	130 (120–138)	123 (106–135)	130 (120–138.25)	**0.016**
ΔHb	7 (1–13)	11 (4–18)	6 (0–12)	**0.005**
TPSA (ng/ml), median (IQR)	3.95 (2.08–7.11)	4.73 (2.66–8.03)	3.98 (2.10–6.87)	0.347
FPSA/TPSA, median (IQR)	0.20 (0.15–0.25)	0.22 (0.17–0.29)	0.20 (0.15–0.25)	0.114
**D-dimer (mg/L), median (IQR)**				
Preoperative	0.15 (0.11–0.24)	0.16 (0.12–0.26)	0.15 (0.10–0.23)	0.123
Postoperative	1.00 (0.47–2.14)	2.53 (1.26–5.62)	0.92 (0.45–2.00)	**0.000**
Postoperative/preoperative	6.59 (3.08–14.04)	11.64 (4.53–27.51)	6.21 (3.00–13.60)	**0.019**
Caprini score, median (IQR)	5 (5–6)	6 (5–8)	5 (5–6)	**0.000**
Operation duration (min), median (IQR)	63.50 (42.75–85.00)	65 (55–100)	60 (45–85)	**0.038**
**IPSS score, median (IQR)**				
Preoperative	22 (20–24)	21 (19–23)	22 (20–24)	0.318
Postoperative	14 (12–15)	14 (12–16)	14 (12–16)	0.678
**QOL score, median (IQR)**				
Preoperative	5 (4–5)	5 (4–5)	5 (4–5)	0.764
Postoperative	2 (1–3)	2 (1–3)	2 (1–3)	0.649
Hypertension	176 (39.02%)	13 (36.11%)	163 (39.27%)	0.709
Diabetes Mellitus	54 (11.97%)	8 (22.22%)	46 (11.08%)	**0.049**
History of surgery (<1 mon)	26 (5.76%)	5 (13.89%)	21 (5.06%)	**0.030**
History of VTE	10 (2.22%)	5 (13.89%)	5 (1.20%)	**0.000**
Family history of VTE	6 (1.33%)	1 (2.78%)	5 (1.20%)	0.431
Postoperative bladder hematoma	29 (6.43 %)	10 (27.78%)	29 (4.5%)	**0.000**

*VTE, venous thromboembolism; BMI, body mass index; Hb, Hemoglobin; TPSA, total prostate specific antigen; FPSA, free prostate specific antigen; IPSS, international prostate symptom score; QOL, quality of life*.

*Note: Some independent variables without statistical significance are not presented in this table, including preoperative hematuria, preoperative urinary tract infections, arthritis, lower limb fracture, lower limb varicose veins, preoperative history of anticoagulant, COPD, heart failure, coronary heart disease, history of tumor, history of cerebral infarction and anemia. Bold values means statistically significant*.

### Baseline Demographic and Clinical Characteristic

The baseline demographic and clinical characteristics of the patients are displayed in Table 1. There were no statistical differences in body mass index (BMI), prostate volume, creatinine, total prostate-specific antigen (TPSA), free prostate-specific antigen (FPSA)/(TPSA), preoperative D-dimer, international prostate symptom score (IPSS), quality of life (QOL) score, hypertension, and family history of VTE between the VTE group and non-VTE group. Meanwhile, some other potential risk factors were showed differences significantly between VTE patients and non-VTE patients, such as postoperative hemoglobin (Hb), postoperative D-dimer, postoperative/preoperative D-dimer, Caprini score, operation duration, diabetes mellitus, history of surgery (<1 month), history of VTE, and postoperative bladder hematoma (Table 1).

### Univariate Logistic Regression Analysis

Univariate logistic regression analysis was further performed to detect the risk factors associated with VTE following TURP. We found that older than 65 years of age, postoperative Hb ≤ 110 g/L, decreased value of Hb in surgery >10.5 g/L, postoperative D-dimer >1.25 mg/L, postoperative/preoperative D-dimer >5, Caprini score >6, surgical duration of >60 min, having a history of surgery within 1 month, having a history of VTE and complicating with a postoperative bladder hematoma were possible risk factors of VTE (Table 2).

**Table 2:** Univariate logistic regression analysis of risk factors associated with VTE following TURP.

	**VTE (*n* = 36)**	**Non-VTE (*n* = 415)**	***P*-value**	**Exp (B)**	**Exp (B) 95% CI**
Age (years)			**0.039**	2.582	1.050–6.350
≤ 65	6	141			
>65	30	274			
Postoperative Hb (g/L)			**0.005**	3.069	1.391–6.769
>110	26	367			
≤ 110	10	46			
ΔHb (g/L)			**0.004**	2.793	1.401–5.566
≤ 10.5	16	286			
>10.5	20	128			
Postoperative D-dimer			**0.000**	4.515	1.951–10.445
≤ 1.25	8	152			
>1.25	24	101			
Postoperative/preoperative D-dimer			**0.042**	2.383	1.031–5.507
≤ 5	8	112			
>5	24	141			
Intraoperative blood loss (ml)			**0.000**	3.970	1.900–8.295
≤ 35	11	262			
>35	25	150			
Caprini score			**0.000**	4.346	2.096–9.014
≤ 6	22	362			
>6	14	53			
Surgery duration (min)			**0.020**	2.385	1.144–4.974
≤ 60	11	212			
>60	25	202			
Diabetes Mellitus	8 (22.22%)	46 (11.08%)	0.054	2.292	0.986–5.327
History of surgery (<1 mon)	5 (13.89%)	21 (5.06%)	**0.038**	3.018	1.065–8.553
History of VTE	5 (13.89%)	5 (1.20%)	**0.000**	13.226	3.633–48.154
Postoperative bladder hematoma	10 (27.78%)	29 (4.5%)	**0.000**	8.016	3.384–18.990

*VTE, venous thromboembolism; ΔHb, preoperative hemoglobin—postoperative hemoglobin*.

*Bold values means statistically significant*.

### Multivariate Logistic Regression Analysis and Nomogram Prediction Model

**Figure 1:**
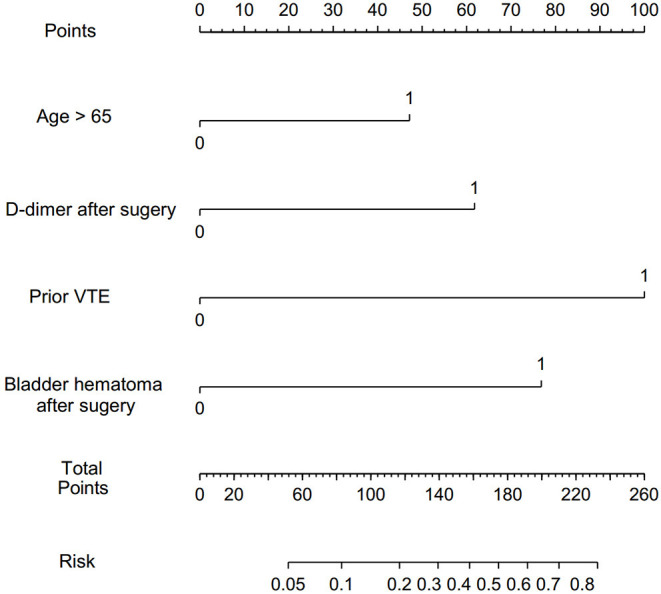
Nomogram to predict the probability of VTE after TURP. VTE, venous thromboembolism; TURP, transurethral resection of the prostate.

To determine the independent effects of risk factors for postoperative VTE following TURP, the multivariate logistic regression and multivariate stepwise logistic regression analyses were performed. In multivariate logistic regression analysis, history of VTE (adjusted odds ratio [aOR] = 8.597, 95% CI = 1.468–50.348), complicating with postoperative bladder hematoma (aOR = 4.250, 95% CI = 1.293–13.971), and D-dimer >1.25 mg/L (aOR = 4.558, 95% CI = 1.413–14.699) were found to be the independent risk factors of VTE after TURP. Following this, the multivariate stepwise logistic regression analysis was performed. Considering that age is an important risk factor for VTE, we took age into consideration in multivariate stepwise logistic regression analysis. The results showed that history of VTE (aOR = 10.980, 95% CI = 2.265–53.223), complicated with postoperative bladder hematoma (aOR = 6.302, 95% CI = 2.265–17.532), D-dimer >1.25 mg/L (aOR = 4.402, 95% CI = 1.798–10.774), and age >65 (aOR = 3.106, 95% CI = 1.084–8.893) were independent risk factors of VTE underwent TURP (Table 3). Furthermore, a nomogram prediction model was established to calculate the cumulative probability of VTE according to the results of multivariate stepwise logistic regression analysis (Figure 1). The total score is the sum of the scores of the four risk factors (age > 65, D-dimer > 1.25 mg/L, history of VTE, and postoperative bladder hematoma), and the VTE risk rate corresponds to the corresponding total score. The predictive ability of the model was evaluated by the C-index (0.793, 95% CI 0.719–0.867, *p* < 0.0001).

**Table 3:** Multivariate and multivariate stepwise logistic regression analysis of risk factors associated with VTE following TURP.

**Risk factors**	**Multivariate logistic regression**			**Multivariate stepwise logistic regression**		
	***P*-Value**	**aOR**	**95% CI**	***P-*value**	**aOR**	**95%CI**
Diabetes Mellitus	0.238	1.892	0.656–5.453			
History of VTE	**0.017**	8.597	1.468–50.348	**0.003**	10.980	2.265–53.223
History of surgery (<1 mon)	0.417	1.777	0.444–7.111			
Postoperative bladder hematoma	**0.017**	4.250	1.293–13.971	**0.000**	6.302	2.265–17.532
Age > 65 (years)	0.097	2.506	0.847–7.417	**0.035**	3.106	1.084–8.893
D-dimer > 1.25 (mg/L)	**0.011**	4.558	1.413–14.699	**0.001**	4.402	1.798–10.774
Postoperative Hb (g/L)	0.914	1.068	0.320–3.571			
Postoperative/preoperative D-dimer	0.876	0.908	0.272–3.037			
Caprini score > 6	0.651	1.279	0.441–3.711			
Operation duration > 60(min)	0.541	1.321	0.541–3.227			
Hemoglobin decline (g/L)	0.218	1.744	0.720–4.224			

*Bold values means statistically significant*.

## Discussion

Venous thromboembolism is a common and potentially fatal complication that happened after surgery, and it has attracted more and more attention in recent years. In this research, we found that the incidence of VTE following TURP was 8.0%, the incidence of DVT was 2.7%, the incidence of PE was 0.4%, and 4.9% for superficial vein thrombosis. This finding was surprising. Because the previous study reported that the incidence of VTE after urological non-oncological surgery was maintained at a range of 0.5–1.4% (13–17). Thus, the urologist seems to pay little attention to VTE in patients after TURP. However, the incidence of VTE in recent years is not as low as reported. The results of our study showed that the incidence of VTE is much higher than previously reported. This may be due to the high postoperative ultrasound screening rate (60%) in our urology department. While, in another research, ultrasonography or venous angiography was performed when patients exhibited VTE-related symptoms or fibrinogen elevation (18, 19). This suggests that many asymptomatic patients with VTE may have been missed. Another reason that caused the high incidence of VTE in our study may be the high proportion of aged patients. The median age in our study was 68 years old and the VTE group patients' median age was 70 years old. We found that age over 65 was an independent risk factor of VTE through the multivariate stepwise logistic regression analysis. This is ~10 years earlier than the age of 75 indicated by the EAU (20). We speculate that this is caused by the lower proportion (20%) of the older patients (>75 years old) in our cohort. As we all know, advanced age is an important risk factor for VTE. Borow et al. reported that the incidence of DVT in patients aged 40–60, older than 71 were 20 and 62.5%, respectively. Though the incidence of DVT was maintained at a low rate among patients who have received thromboprophylaxis therapy, the incidence doubled in patients aged 60–70 years old and increased three times in patients older than 71 (21). Naess et al. revealed that the incidence of VTE increased exponentially with age. Incidence in subjects aged 70 years or above was more than three times higher than those in subjects aged 45–69 years, which again were three times higher than the incidence in subjects aged 20–44 years (22). There are many reasons for elderly patients prone to suffer VTE. An abnormal high level of coagulation factors in the blood is a possible reason (23). Meanwhile, Koster's research recommended that high factor VIII concentrations represent a clear increase in the risk of thrombosis (24). Other possible reasons include poor mobility, lower cardiorespiratory fitness, and more complications. D-dimer is the simplest fibrin degradation product. An increased level of D-dimer indicates a hypercoagulable state in the blood. So, in our department, a D-dimer test was performed routinely to detect VTE due to its high sensitivity. The results of this study showed that the level of D-dimer >1.25 mg/L was an independent risk factor. The critical value of D-dimer was much higher than previously reported (6, 25). This difference may result from the high proportion of aged patients in this study. As is known to all, D-dimer level was not only increased in most of the patients with VTE but the level was also influenced by several other factors, such as aging, surgery, cardiovascular diseases, and so on (26–28). To reduce the influence of advanced age on the diagnostic specificity of D-dimer, some researchers have proposed to adjust the threshold of D-dimer to predict VTE based on age (age × 10 μg/L) (29). Moreover, the studies of Schouten and Han have confirmed that the age-adjusted cut-off of D-dimer values can improve its diagnostic specificity without affecting sensitivity (30, 31). So, in elderly patients after TURP, a mild elevated D-dimer (<1.25 mg/L) may not be considered as a risk factor, but they are still recommended to perform D-dimer test after TURP owing to its high sensitivity. In this study, we found that having a history of VTE was an independent risk factor of VTE, which is consistent with previous studies (32, 33). Meanwhile, the American College of Chest Physicians (ACCP-9), EAU, and American Urological Association (AUA) confirmed that the history of VTE was an important risk factor (20, 34, 35). So, prior VTE history should be emphasized in risk stratification for patients after TURP. Postoperative bladder hematoma was a risk factor of VTE following TURP according to the multivariate stepwise logistic analysis. If the color of the continuous bladder irrigation is bright red and combined with a decreased Hb after TURP, the bladder ultrasound will be performed to confirm whether there is a bladder hematoma. Patients with bladder hematoma after TURP have a significantly increased risk of VTE. This may be due to the longer time spent in bed for these parts of patients. Furthermore, bleeding can lead to activation of the coagulation system and made the blood of these patients in a hypercoagulable state. Intriguingly, there was no significant difference in the Carprini score between the VTE group and the non-VTE group in our cohort (after the multivariate stepwise logistic regression analysis). Age over 60 is defined as 2 points and surgery is defined as 1 or 2 points according to the Caprini risk assessment model (RAM). However, most of the patients in our study were older than 60. So, their baseline Caprini score was at least 3 points (age >60 and underwent TURP). This means that most of these patients were at moderate risk of VTE in the Caprini RAM. Another indicator without significant difference is BMI. This is because it is not easy to find elderly individuals whose BMI was more than 35 in our department. The highest BMI was 33.65 among all patients included in this study. Further studies on these two factors are needed. Compared to transurethral resection of bladder tumor (TURBT), we speculate that the development of VTE following TURP may be related to elderly age, the prolonged lithotomy position procedure, longer duration of bed rest after surgery, higher risk of bleeding, etc. Prolonged lithotomy position procedure may lead to poor lower extremity venous reflux and alter hemodynamic states. This may increase the risk of thrombosis. In addition, perioperative bleeding can activate the coagulation system cause a higher possibility of VTE. However, as for TURBT, the main risk factor of VTE is the tumor. Various studies have shown that the tumor can obviously increase the risk of VTE (36, 37). As far as we know, this is the first cohort study specifically focusing on VTE after TURP. We established a nomogram prediction model of VTE for patients who underwent TURP. It is an auxiliary prevention tool of VTE beneficial to clinical practice. However, we must propose that there are some limitations to this study. First, this is a single-center retrospective study. Second, the number of patients included in this study is small. Third, the period of observation for patients with VTE was only during hospitalization. The long-term follow-up for discharged VTE patients was still needed. Considering the limitations of our study, further large-scaled and multicenter studies are urgently needed to confirm the result.

## Conclusion

The incidence of VTE is severely underestimated in patients following TURP. A lot of asymptomatic VTEs have been overlooked. On the other hand, history of VTE, postoperative bladder hematoma, age >65 (years), and D-dimer >1.25 (mg/L) are independent risk factors. So, it is important that VTE is detected and diagnosed early in patients after TURP according to these risk factors. Nevertheless, further verifications based on the results of large-scaled studies are still needed.

## Data Availability Statement

The raw data supporting the conclusions of this article will be made available by the authors, without undue reservation.

## Author Contributions

ZT and ZWa conceived and designed the study. ZZ, ZWu, KL, QZ, HL, XL, and GW collected the data. ZZ, ZWu, and ZWa analyzed and were involved in the interpretation of data. ZZ and ZWu drafted the manuscript. ZT, ZWa, ZZ, and ZWu made final approval of the version to be published. All authors contributed to the article and approved the submitted version.

## Funding

This work was supported by the Project from the National Health Commission of Hunan Province to Tang Zhengyan (B2019185).

## Conflict of Interest

The authors declare that the research was conducted in the absence of any commercial or financial relationships that could be construed as a potential conflict of interest.

## Publisher's Note

All claims expressed in this article are solely those of the authors and do not necessarily represent those of their affiliated organizations, or those of the publisher, the editors and the reviewers. Any product that may be evaluated in this article, or claim that may be made by its manufacturer, is not guaranteed or endorsed by the publisher.
